# Development of Nafamostat Mesylate Immediate-Release Tablet by Drug Repositioning Using Quality-by-Design Approach

**DOI:** 10.3390/pharmaceutics14061219

**Published:** 2022-06-08

**Authors:** Hyeon-A Kim, Joo-Eun Kim

**Affiliations:** Department of Pharmaceutical Engineering, Catholic University of Daegu, Hayang-Ro 13-13, Gyeongsan City 38430, Korea; missha5122@naver.com

**Keywords:** nafamostat mesylate, immediate-release tablets, COVID-19, quality-by-design approach, drug repositioning, pharmacokinetic study

## Abstract

We aimed to develop nafamostat mesylate immediate-release tablets for the treatment of COVID-19 through drug repositioning studies of nafamostat mesylate injection. Nafamostat mesylate is a serine protease inhibitor known to inhibit the activity of the transmembrane protease, serine 2 enzyme that affects the penetration of the COVID-19 virus, thereby preventing the binding of the angiotensin-converting enzyme 2 receptor in vivo and the spike protein of the COVID-19 virus. The formulation was selected through a stability study after manufacturing by a wet granulation process and a direct tableting process to develop a stable nafamostat mesylate immediate-release tablet. Formulation issues for the selected processes were addressed using the design of experiments and quality-by-design approaches. The dissolution rate of the developed tablet was confirmed to be >90% within 30 min in the four major dissolutions, except in the pH 6.8 dissolution medium. Additionally, an in vivo pharmacokinetic study was performed in monkeys, and the pharmacokinetic profiles of nafamostat injections, oral solutions, and tablets were compared. The half-life during oral administration was confirmed to be significantly longer than the reported literature value of 8 min, and the bioavailability of the tablet was approximately 25% higher than that of the oral solution.

## 1. Introduction

The Coronavirus Disease 19 (COVID-19) virus first originated in Wuhan City, Hubei Province, in December 2019; it has now spread worldwide as a respiratory infectious disease. COVID-19 was confirmed to be an RNA virus belonging to *Coronaviridae*, and, by analyzing the gene sequence of the virus, it was confirmed to have the highest similarity to the bat-derived coronavirus [1,2,3,4,5]. The infection route of COVID-19 involves introduction and infection in vivo when transmembrane protease, serine 2 (TMPRSS2) is cleaved after the spike protein of the COVID-19 virus binds to the angiotensin-converting enzyme 2 receptor. A recent study confirmed that TMPRSS2 protease is related to the spread of COVID-19 and disease occurrence. Accordingly, a drug that inhibits TMPRSS2 activity is highly likely to be an effective therapeutic drug for COVID-19. In addition, when the activity of TMPRSS2 was blocked, membrane fusion between the virus and human cells was inhibited, virus invasion was blocked, and the pathogenicity and infectivity of the virus could be minimized by anti-plasmin activity [6,7,8,9,10,11]. Among the drugs that strongly inhibit TMPRSS2, nafamostat mesylate has the strongest antiviral effect against COVID-19, and its IC_50_ value is approximately 600 times higher than that of remdesivir, the standard treatment for COVID-19 [12]. Thus, nafamostat mesylate was determined to be a suitable drug for drug repositioning studies. Drug repositioning is the identification of new indications for drugs that are already on the market or that have failed in clinical trials, and it has the advantage of reducing costs and time by approximately 25–35% compared to the development of existing new drugs [13,14,15,16,17,18]. 

In the case of drugs used for drug repositioning, the mechanism of action of the drug is widely known, and, depending on this, it can be divided into on-target and off-target strategies. An on-target strategy is a novel approach for finding the target–disease relationship. It is a method of repositioning that involves analyzing the applicability of the target for other indications. Furthermore, it involves finding new indications for the drugs by identifying the relationship between the drug targets and new diseases and investigating novel applications for them. In contrast, the off-target strategy is based on a new drug–new target relationship and is an approach to identifying novel applications for drugs by screening drugs through various screening systems to find the relationship with the new drug targets. As a result, the development of a therapeutic agent is achieved through a target different from the initial target of the drug [19,20,21,22].

In the case of nafamostat, it was initially used as a protease inhibitor during blood coagulation; however, it was later found that it inhibits the TMPRSS2 protease during coronavirus infection and, therefore, corresponds to an off-target strategy. Moreover, through a literature search related to the repositioning of nafamostat, we confirmed that nafamostat strongly inhibits TMPRSS2 [23,24,25,26,27]. Accordingly, we selected nafamostat mesylate for drug repositioning, which strongly inhibits TMPRSS2 protease and can reduce the period and cost of development. However, nafamostat mesylate has a short half-life of 8 min, poor water stability, and low solubility at specific pH, which makes it difficult for tableting. Therefore, currently marketed formulations have only been developed in the form of injections. Since the half-life is very short, the current dosage method uses an intravenous injection approach by dissolving 20–50 mg every hour in a 5% glucose injection solution. Therefore, it might be possible to improve the ease of administration by patients by changing the injection into a tablet formulation. For preparing the optimal formulation of nafamostat, a manufacturing method was selected by comparing wet and direct compression processes. Moreover, we aimed to choose a method that focuses on studying the stability of the tablet [28,29,30]. To develop a formulation study of nafamostat, we applied QbD approaches, which involve building high-quality drug products by statistical design. The QbD approach ensures that the pharmaceutical development process is focused on providing a scientific understanding of how material and process parameters affect drug product performance. This knowledge enables reasonable manufacturing controls that ensure the consistent accomplishment of all necessary quality targets, formulation design, and drug product requirements [31]. In addition, we attempted to confirm the bioavailability of the final formulation by calculating the maximum concentration (C_max_) and area under the curve (AUC) in a monkey pharmacokinetic (PK) study. In addition, it is anticipated to improve the convenience of administration of existing anticoagulants in pancreatitis patients due to the change in the dosage form.

## 2. Materials and Methods

### 2.1. Materials

The active pharmaceutical ingredient (API), nafamostat mesylate, was purchased from Kukjeon (Anyang, Gyeonggi-do, Korea). Microcelac was supplied by Meggle (Wasserburg am Inc., Upper Bavaria, Germany). Crospovidone was supplied by BASK (Carl-Bosch-Str.38; Ludwigshafen, Germany). Hydroxypropyl cellulose was purchased from Nippon Soda (Chiyoda-ku, Tokyo, Japan). Sodium stearyl fumarate was purchased from JRS Pharma (Holzmuhle 1, Rosenberg, Germany). The following reagents were used throughout the study: microcrystalline cellulose (Heweten 102, JRS Pharma, Rosenberg, Germany), lactose (Pgarmatose 200M, DFE Pharma, Goch, Germany), mannitol (Pearlitol 200SD, Roquette Pharma, Lestrem, France), dicalcium phosphate dihydrate (Innophos, Cranbury, NJ, USA), pregelatinized starch (Starch 1500, Colorcon, Shanghai, China), hydroxypropyl methylcellulose (METHOCEL, Colorcon, Shanghai, China), sodium starch glycolate (GLYCOLYS, Roquette, Lestrem, France), magnesium stearate (Faci Asia Pacific Pty Ltd., Jurong Island, Singapore), and croscarmellose sodium (VIVASOL, JRS Pharma, Rosenberg, Germany). All other chemicals were of analytical reagent grade and were purchased commercially.

### 2.2. Solubility Studies

The solubility of the API was evaluated in deionized water (D.I.W.), pH buffers (pH 1.2–12.0), and organic solvents (ethanol and methanol). In the apparent solubility test, approximately 10 mg of nafamostat mesylate was added to each vial, and 200 µL of the solvent was added and stirred. The approximate solubility was calculated by adding 200 µL of solvent until it was clear to the naked eye and recording the volume of solvent added. In the equilibrium solubility test, the excess drug was added to each vial, which was reflected by the apparent solubility test, and stirred at 400 rpm. A certain amount was collected at 1 h and 4 h, filtered through a 0.45 µm RC syringe filter, and then diluted to the same concentration as the standard solution. The maximum solubility of each solvent was calculated using the high-performance liquid chromatography (HPLC) method described in Section 2.6.1.

### 2.3. Compatibility Studies between API and Excipients

To confirm whether nafamostat mesylate and each excipient interacted, nafamostat mesylate was mixed with the following excipients in a 1:1 ratio (*w*/*w*): mannitol, lactose, dicalcium phosphate, microcrystalline cellulose, pregelatinized starch, precipitated calcium carbonate, sodium starch glycolate, crospovidone, sodium croscarmellose, sodium stearyl fumarate, magnesium stearate, povidone K, hydroxypropyl methylcellulose, calcium hydroxide, and hydroxypropyl cellulose. The mixed sample was stored for 4 weeks in a stability chamber under conditions of 40 ± 2 °C/75 ± 5% RH and 60 ± 2 °C/95 ± 5% RH. The interaction between the active ingredient and excipient was evaluated by appearance, differential scanning calorimetry (DSC, DSC Q2000, TA Instruments, New Castle, DE, USA), and impurity analysis. DSC analysis confirmed the change in the endothermic exothermic peak upon heating 1 to 3 mg of the mixed sample at a rate of 10 °C/min from 25 to 330 °C. The impurities were analyzed by HPLC (Agilent 1260 Infinity II, Agilent Technologies, La Jolla, CA, USA) according to the Related Substances section of the purity section of nafamostat mesylate present in the Japanese Pharmacopoeia.

### 2.4. Formulation of Nafamostat Mesylate

After selecting an excipient in consideration of the compatibility studies, it was manufactured by a wet granulation process and a direct tableting process to develop a long-term stable and optimized formulation. A relatively stable formulation was selected through a 2 month stability test. For the wet granulation process, a high-speed mixer (YC-SMG-3, YENCHEN MACHINERY Co., Ltd., Taoyuan City, Taiwan) was used, the granules were prepared, and the impeller and chopper speeds were maintained at 150 and 1600 rpm for 3 min, respectively. The dried granules were sieved with a 16-mesh sieve and then mixed with sodium stearyl fumarate as a lubricant to prepare the final mixture. In the direct tableting process, after mixing microcelac, crospovidone, and hydroxypropyl cellulose, sodium stearyl fumarate was used as the lubricant to prepare the final mixture. Both formulations were compressed to approximately 12 kN using a rotary compression machine (PR-3000 Series; PTK, Gimpo City, Korea). A relatively stable formulation was selected on the basis of a stability study of the two formulations.

### 2.5. Critical Quality Attributes (CQA)/Risk Assessment (RA) of Critical Material Attributes (CMA) and Critical Process Parameters (CPP)

**Figure 1:**
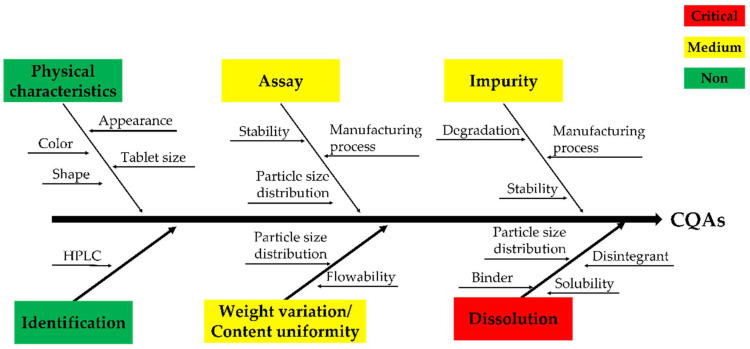
Fishbone/Ishikawa diagram regarding the critical quality attributes (CQAs).

It was difficult to determine the hardness appropriately when developing the formulation with the process selected through the stability study; therefore, as shown Figure 1, we used the QbD approach to identify the CQA [32,33,34]. To identify CQA, quality characteristics such as physical characteristics, identification, assay, weight variation/content uniformity, impurities, and dissolution were designated. RA was performed by selecting the CMA and CPP that affect the CQA. RA is a tool that can identify all potential variables that can affect quality [29]. Moreover, process hazard analysis (PHA) and failure modes and effects analysis (FMEA) were used as performance tools [35]. PHA is a preliminary hazard analysis, which refers to RA in the preparatory stage before full-scale RA, and the level of risk must be controlled to an acceptable level during production [36]. The PHA indicates the level of risk for each item in green, yellow, and red colors. Green represents a broadly acceptable level of risk, and yellow represents an acceptable level of risk; therefore, further research and feasibility may be needed to reduce the risk. Lastly, red represents an unacceptable level of risk, indicating that additional research is essential to reduce the risk. FMEA is a tool that can increase product reliability by evaluating potential failure modes and their impact on output or product performance during product and process production [37]. The evaluation items consisted of probability (P), severity (S), and detectability (D). P is the probability-based failure score, S is based on the impact of the CQA severity, and D is the probability and severity of undetected failures. The score was calculated from the lowest score of one point to the highest score of four points. The risk of failure is expressed as risk priority number (RPN), which is the product of P, S, and D, and it was calculated on the basis of previous experience and a literature review [38,39,40]. If the total RPN value is 30 or higher, major measures such as DoE should be taken in preparation for risk. The binder and disintegrant were selected as the CQA through RA, and the design space was derived using the central composite design of the DoE.

### 2.6. HPLC Analysis

#### 2.6.1. Assay

The nafamostat mesylate assay method was modified and referred to in the related substance section of the purity of nafamostat mesylate drug substance present in the Japanese Pharmacopoeia [41]. The assay of nafamostat mesylate was analyzed using HPLC (Agilent 1260 Infinity II, Agilent Technologies, USA) equipped with an HPLC separation module and a UV detector. For preparing the mobile phase, 6.07 g of sodium 1-heptane sulfonate was dissolved in 6 mL of acetic acid, and water was added to obtain a total volume of 1000 mL. Acetonitrile (ACN; 300 mL) was added to 700 mL of this solution, mixed with a 0.22 µm membrane filter, and further filtered. The solution was then degassed using a degassing device. The column used was Sunfire^®^ C18 (4.6 × 250 mm, 5.0 µm), and the column temperature and injection volume were set at 40 °C and 10 µL, respectively. The flow rate was adjusted such that the retention time of nafamostat mesylate was approximately 7 min. The peak measuring wavelength was 260 nm, and the measuring range was approximately twice that of the retention time of nafamostat mesylate. The calibration sample was prepared by accurately weighing 100 mg of nafamostat, putting it in a 100 mL volumetric flask, and dissolving it in a mobile phase to obtain a stock solution, which was then diluted to a concentration of 0.02 to 0.4 mg/mL.

The calibration curve was linear with a correlation coefficient of 0.999, and the standard deviation of the accuracy and precision was within 2%.

#### 2.6.2. Impurity

Nafamostat mesylate impurity assay was modified according to the related substance section of the purity of the nafamostat mesylate drug substance present in the Japanese Pharmacopoeia. The specifications for impurities were set at 0.2% or less of individual impurities, 0.2% of unknown impurities, and 2.0% or less of total impurities, referring to the related substances section in the Japanese Pharmacopoeia nafamostat mesylate and ICH guideline Q3B [41,42]. The impurity of nafamostat mesylate was analyzed using HPLC (Agilent 1260 Infinity II, Agilent Technologies, USA) equipped with an HPLC separation module and a UV detector. For the mobile phase, 6.07 g of sodium 1-heptane sulfonate was dissolved in 6 mL of acetic acid, and water was added to obtain a total volume of 1000 mL. ACN (300 mL) was added to 700 mL of this solution. The column used was Sunfire^®^ C18 (4.6 × 250 mm, 5.0 µm), and the column temperature and injection volume were set at 40 °C and 10 µL, respectively. The flow rate was adjusted such that the retention time of nafamostat mesylate was approximately 7 min. The peak measuring wavelength was 260 nm, and the measuring range was approximately four times that of the retention time of nafamostat mesylate.

### 2.7. Stability Studies

To select effective and stable immediate-release tablets of nafamostat mesylate, the tablets from both the wet granulation process and the direct tableting process were stored for 2 months in a stability chamber maintained at 25 ± 2/60 ± 5% RH, 40 ± 2 °C/75 ± 5% RH, and 60 ± 2 °C/95 ± 5% RH. A stability study was conducted using this method. The specification and test methods for impurities in nafamostat mesylate were established by referring to the related substances section in the Japanese Pharmacopoeia and ICH guideline Q3B. The impurities in the two tablets were analyzed using the HPLC method described in Section 2.6.2.

### 2.8. In Vitro Dissolution Studies

The in vitro dissolution test was performed according to the USP <711> Dissolution Apparatus 2 (Paddle apparatus) using a dissolution tester (KDT1200, Kukje Engineering, Goyang, Korea) [43]. For the dissolution medium, 900 mL of pH 1.2, 4.0, 6.8, and D.I.W. was used, the temperature of the dissolution medium was set to 37.0 ± 0.5 °C, and the speed of the paddle was set to 75 rpm. After the initiation of dissolution, tablets were added, and the mixture was incubated for 1 h. The collected sample was filtered with a 0.45 µm RC syringe filter as the sample solution. The sample solution was analyzed by HPLC (Agilent 1260 Infinity II, Agilent Technologies, USA), and the wavelength of the UV detector was 260 nm. The column used was Sunfire^®^ C18 (4.6 × 250 mm, 5.0 µm).

### 2.9. Pharmacokinetics Studies

The PK study confirmed the pharmacokinetic profile of nafamostat mesylate immediate-release tablets in monkeys. Seven male monkeys were used in the study to evaluate the pharmacokinetics of the tablet. All procedures for animal testing were in compliance with the Animal Welfare Act and guidelines for the protection and use of laboratory animals. This study was approved by the Animal Care and Use Committee of ORIENT BIO Inc., Seongnam, Korea (ORIENT-IACUC-20133). Nafamostat injection was used as the reference drug, and a nafamostat solution and immediate-release tablet were used as the test drug. The nafamostat solution was used at the same dose as that of the immediate-release tablet. For the reference drug (nafamostat injection) and test drug (nafamostat solution and immediate-release tablet), two reference drugs, two solutions, and three immediate-release tablets were divided into three groups and allowed to fast for approximately 12 h. The blood sampling times of the reference drug were 0.01, 0.03, 0.1, 0.3, 0.5, 1, 2, and 4 h after intravenous injection, and the test drug (liquid and immediate-release tablet) was administered orally at 0.5, 1, 2, 3, 4, 6, and 8 h. Approximately 500 µL of blood was collected using a heparinized syringe. The collected blood was centrifuged at 4000 rpm for approximately 10 min to separate plasma. The separated plasma samples were frozen and stored below −70 °C until analysis. Normalized liquid chromatography–tandem mass spectrometry (QTRAP^®^ 4500 LC-MS/MS System, AB SCIEX Pte., Ltd., Framingham, MA, USA) was used for the analysis of plasma nafamostat concentration, and pharmacokinetic analysis was performed using Phoenix WinNonlin™ software (ver. 8.2, CERTARA, Princeton, NJ, USA) in a noncompartmental manner.

### 2.10. Statistical Analysis

Data are expressed as the mean (x) ± SD. Mean comparisons between the groups were performed using single-factor analysis of variance, and LSD tests were used for pairwise comparisons. Differences were considered statistically significant at *p* ≤ 0.05. The Minitab^®^ software program (ver. 18, Minitab^®^ Inc., State College, PA, USA) was used for all the statistical analyses. Noncompartmental analyses to derive pharmacokinetic parameters were performed using Phoenix WinNonlin™ software (ver. 8.2, CERTARA, Princeton, NJ, USA). AUC_last_ is the AUC from time 0 to the time of measurable concentration and was calculated by applying the trapezoidal formula. C_max_ is the highest blood concentration, which is the highest blood concentration measured for a given time. T_max_ is the time required to reach C_max_. The equivalence of the nafamostat injection and immediate-release tablets was determined by the individual C_max_ and AUC values derived using log-transformed data and their ratios. ANOVA was used to analyze the mean and 90% confidence interval (CI), and two-way ANOVA was used as the parameter. The effects of the agents, duration, and sequence on pharmacokinetic parameters were evaluated as fixed effects, and the effects of subjects nested within sequences were evaluated as random effects. Another main effect was the error term, which was tested at a 5% significance level for the residual error of the ANOVA model. This test is equivalent to performing two one-sided *t*-tests, similar to the CI of normal theory. If the geometric mean ratio and 90% confidence interval of the C_max_ and AUC of the test drug and the reference drug were within 0.8–1.25 of the log-transformed data range, the two drugs were judged to be equivalent.

## 3. Results and Discussion

### 3.1. Physicochemical Properties of Nafamostat Mesylate

As shown in Table 1, nafamostat mesylate has good solubility. Due to its pKa and log *p* values, it is predicted that absorption from the stomach will be efficient, while the solubility will be poor at a certain pH. Moreover, it is a TMPRSS2 inhibitor with a short half-life of 8 min.

### 3.2. Solubility Studies

The solubility of nafamostat mesylate in various solvents is presented in Table 2. According to the results of the apparent and equilibrium solubility tests, nafamostat mesylate was “slightly soluble” in DI water, as well as pH 2.0, pH 4.0, and pH 8.0 buffers, according to USP and Korean Pharmacopoeia standards. Although the API showed low solubility at pH 1.2 and 2.0, it was understood that there was no major problem as it had sufficient solubility as an immediate-release tablet in 500–1500 mL of gastric juice. As 8.558 mg or more per 1 mL of the API could be dissolved in all dissolution test solutions except in pH 6.8, it was considered highly likely to be a favorable condition for dissolution tests and immediate-release control.

### 3.3. Compatibility Studies between API and Excipients

**Figure 2:**
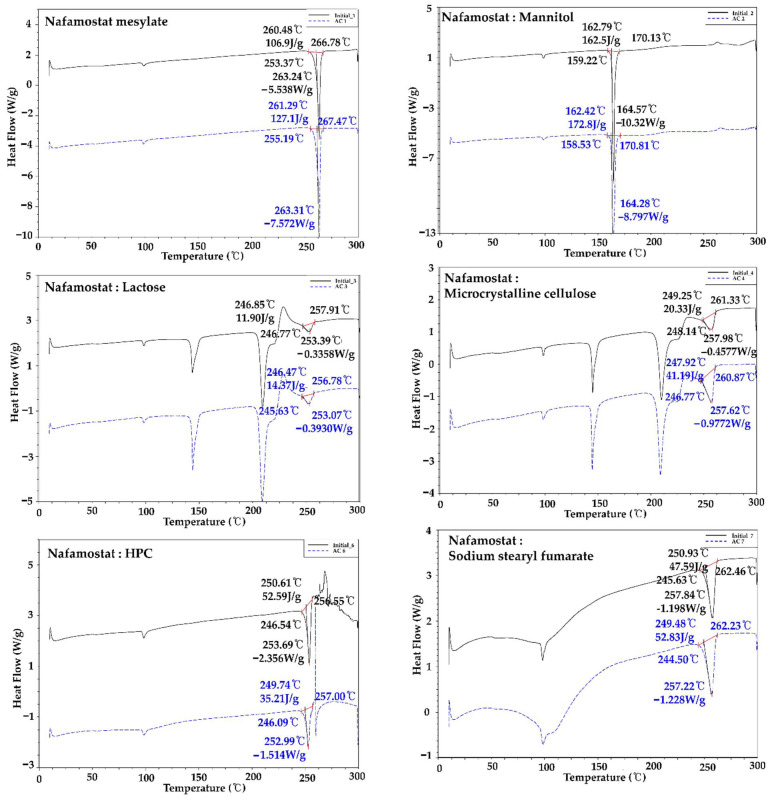

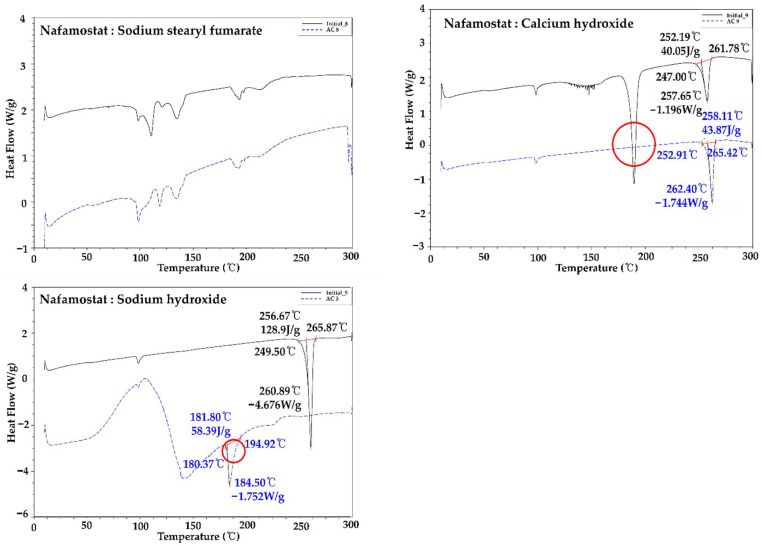
Compatibility test of nafamostat mesylate and each excipient using DSC.

Nafamostat mesylate and excipient were mixed in a 1:1 ratio, and the interaction between the API and excipient was confirmed by DSC and related substance analysis. The melting point range of nafamostat mesylate was found to be about 253 to 267 °C. As a result of the change in appearance after 4 weeks, brown coloration was observed with only two alkylating agents, calcium hydroxide and sodium hydroxide, and, as a result of the DSC change, as shown in Figure 2, a peak shift occurred only in the presence of alkylating agents, calcium hydroxide and sodium hydroxide. This implies that the 1:1 mixture was thermally unstable and was not used in the final formulation because it interacted with excipients at high pH. In addition, as a result of the impurity test in Table 3, rapid impurities were generated in dicalcium phosphate dihydrate, precipitated calcium carbonate, sodium starch glycolate, magnesium stearate, calcium hydroxide, and sodium hydroxide. Therefore, they were not used for the final formulation. As a result of the comprehensive judgment, it was found that nafamostat mesylate was unstable at high pH. Therefore, formulation studies were conducted using the excipients mannitol, crospovidone, sodium stearyl fumarate, and hydroxypropyl cellulose, which showed the lowest impurities except for excipients with high pH.

### 3.4. Stability Studies

The two tablets manufactured according to the wet granulation process and the direct tableting process were stored at 25 ± 2/60 ± 5% RH, 40 ± 2 °C/75 ± 5% RH, and 60 ± 2 °C/95 ± 5% RH. A stability study was conducted along with an impurity test every month, as well as after every 2 months; the results obtained are shown in Table 4(a). The specifications for impurities were set at 0.2% or less for individual impurity, 0.2% for unknown impurities, and 2.0% or less for total impurities, with reference to the related substances section in the Japanese Pharmacopoeia nafamostat mesylate and ICH guideline Q3B. The stability of the wet granulation process was 0.25%, 2.10%, and 9.35% at room temperature, accelerated, and harsh conditions, respectively, and that of the direct tableting process was confirmed to be 0.11%, 0.33%, and 3.04%, respectively. It was concluded that impurities were rapidly generated due to the interaction between the water used as the binding solution and the temperature during drying, owing to the physicochemical characteristics of nafamostat mesylate. In addition, as shown in Table 4(b), it was confirmed that a lower excipient ratio resulted in a more stable system. Finally, to obtain the most stable immediate-release tablet, the ratio of excipients was reduced, and a direct tableting process was selected.

### 3.5. CQA/RA of CMAs and CPPs

Based on the physicochemical properties of the nafamostat mesylate, CQA was selected for dissolution. RA was performed to identify CMA and CPP that affect CQA, and PHA and FMEA were performed for the RA of CMAs and CPPs, according to previous experience and preliminary experiments. As shown in Table 5, the FMEA searching for CMAs was analyzed for failure mode, and the effects on CQAs and RPNs were calculated for each parameter by probability (P), severity (S), and detectability (D).

**Figure 3:**
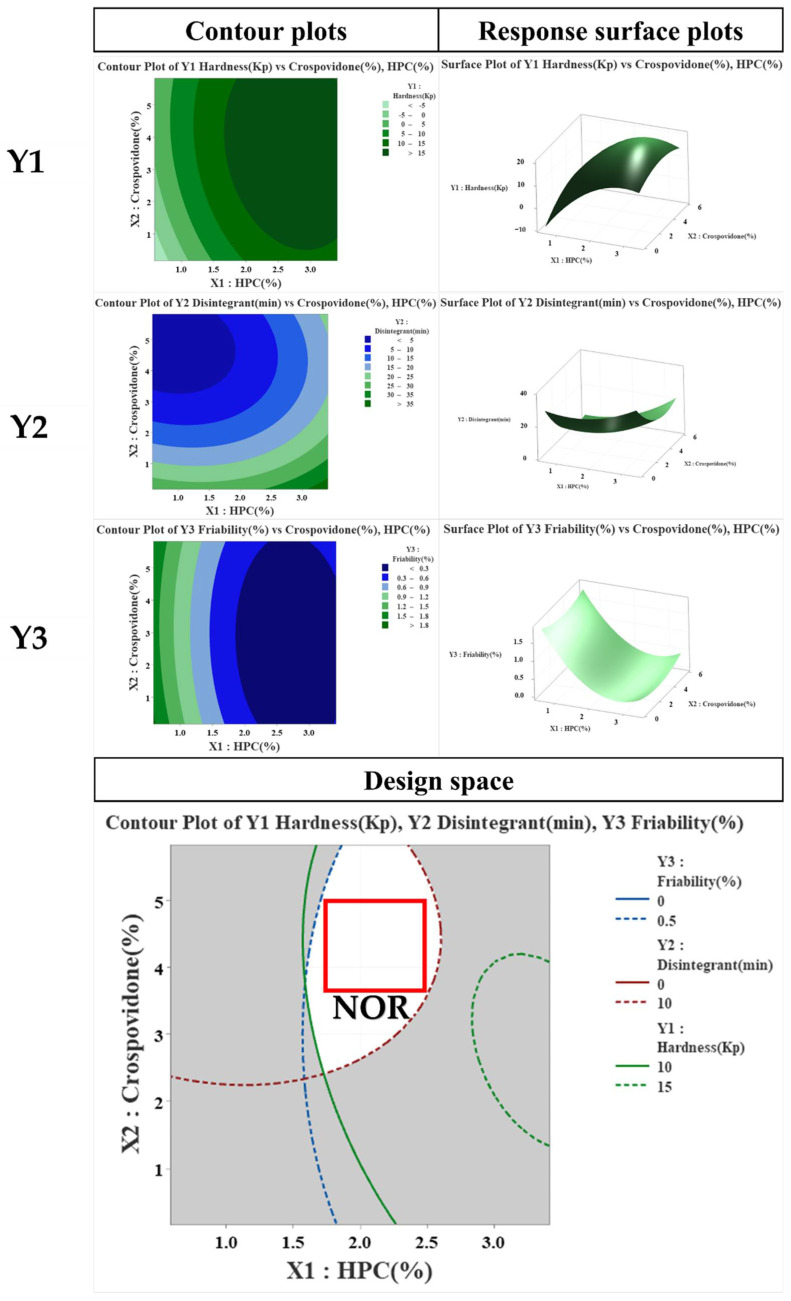
Effects of critical material attributes (CMAs) using contour plots, and response surface plots.

On the basis of these results, the binder and disintegrant were selected as CMA, as shown in Table 6. In contrast, in the case of CPP, PP was not selected because it was understood that it could be sufficiently controlled through prior research and experience. In tablets, binders can affect the timing of the dissolution of the active ingredient. If more than the optimal amount is used, hardness increases during tablet manufacturing, but disintegration and dissolution time delays may occur. In the case of a disintegrant, if a certain amount is not used, not only does the hardness decrease during tablet manufacture, but the desired dissolution pattern also cannot be secured, which may cause damage to effectiveness. Hence, the RPN calculated through FMEA showed that the potential risk is “disintegrant”. Owing to these problems, it was understood that the suitable measures in the experimental design method were necessary to select an appropriate range. As shown in Table 6, two types of CMAs (binder and disintegrant), through the PHA/FMEA of RA, were selected as two input (X) values for independent variables [44]. Using DoE’s central composite design method, the main variable ranges were set to 1.0–3.0% for binder and 1.0–5.0% for disintegrant. As the dependent variables affected by the two X values, hardness, disintegration, and friability were selected. The mathematical response surface models were generated by statistical analysis using the Minitab 18^®^ program by applying coded values of factor levels. Therefore, the results were interpreted after optimizing the DoE modeling. As shown in Figure 3, in the contour and response surface plots for hardness (Y1), we confirmed that, with the increasing amount of HPC, the hardness increased. However, the amount of crospovidone had a minor effect on hardness. In the contour and response surface plots for disintegration (Y2), we confirmed that, with the increasing amount of crospovidone, disintegration time shortened and that, with the increasing amount of HPC, disintegration time increased. Lastly, friability (Y3) showed that HPC rather than crospovidone affected friability. The optimum design space was obtained by overlapping the contour plots of Y1, Y2, and Y3. By overlapping the contour plots of each response value, the ideal window of the working space was confirmed using the design space. To establish the design space, the range of hardness, disintegration, and friability was set to 10.0–15.0 Kp, within 10 min, and within 0.5%, respectively [31]. The design space of the response variables and influencing factors that met the established criteria were verified within a 95% confidence level. The optimized space was designated as a white area; as a result, it was confirmed that the optimized binder and disintegrant composition had hardness, disintegration, and friability values of 12 kP, 6 min, and 0.21%, respectively.

### 3.6. In Vitro Dissolution Studies

**Figure 4:**
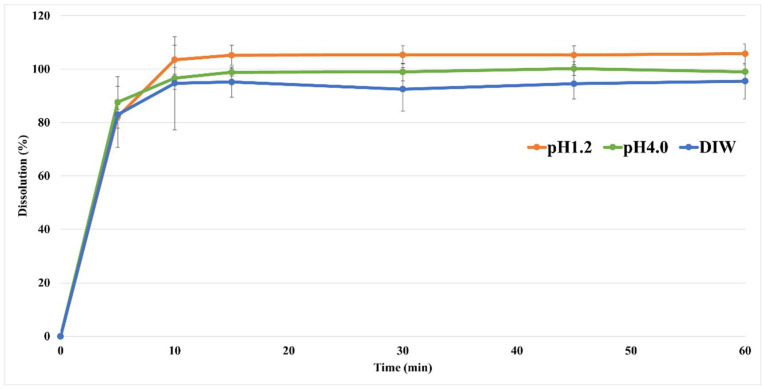
The dissolution profile of nafamostat mesylate from tablets at pH 1.2 and pH 4.0, as well as in DI water.

The dissolution profile is an important indicator to confirm the disintegration and absorption patterns in the human body, which can predict the results of PK and in vivo experiments. An in vitro dissolution study of the nafamostat tablets was performed; the results are shown in Figure 4. At pH 1.2 and 4.0, as well as in DI water, all the tablets showed a pattern with a dissolution rate of 80% or more within 30 min. Therefore, the dissolution rate target of the nafamostat immediate-release formulation was 80% or more in 30 min, and all of them met the target value. In the case of the pH 6.8 buffer, dissolution of the tablet did not occur at a solubility of 0.010 mg/mL, and it could be estimated that dissolution and absorption did not occur in the high-pH region, as in the solubility test result.

### 3.7. Pharmacokinetic Study in Monkeys

**Figure 5:**
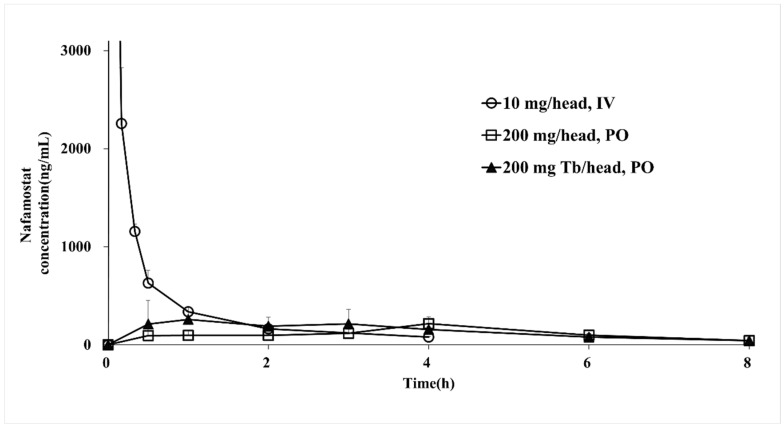
Plasma concentration–time profiles of nafamostat mesylate injections, liquids, and IR tablets.

The bioequivalence of the reference drug (nafamostat injection) and the test drug (nafamostat solution and immediate-release tablet) was evaluated in the PK study. The pharmacokinetic parameters are presented in Table 7, and the PK evaluation is shown in Figure 5. By measuring the nafamostat concentration of the reference drug injected intravenously into monkeys, the C_max_ was found to be 11,108 ng/mL at 0.02 h, and the elimination half-life was identified as 1.5 h. For the drug solution, the C_max_ was 216 ng/mL at 4.00 h, and the blood half-life was determined to be 1.9 h. In the case of the immediate-release tablet, the C_max_ was 327 ng/mL at 1.00 h, and the blood half-life was 2.2 h, which decreased more slowly than the reference drug. The nafamostat blood concentration of the immediate-release tablet was detected for up to 8 h. It was confirmed that the half-life of the immediate-release tablet was the longest among the reference drug and the test drug and was significantly longer than the value reported in the literature [45]. The AUC_last_ of the reference drug was 2183 ng h/mL, the AUC_last_ of the solution formulation was 899 ng h/mL, and the AUC_last_ of the immediate-release tablet was 1141 ng h/mL. When administered orally, the bioavailability was as low as 5% or less. Although the injection had the highest bioavailability, it was confirmed that the immediate-release tablet had better bioavailability than the liquid formulation when administered orally. In addition, the oral formulations showed a PK profile in the form of a sustained-release formulation, and the blood concentration was maintained even after 8 h. A comparison between the oral formulations showed that the bioavailability of the immediate-release tablet was approximately 25% higher than that of the solution formulation. Systemic exposure to nafamostat in terms of C_max_ and AUC_last_ for the three formulations was not statistically significant (*p* > 0.05). According to the results of the dissolution test and PK study of the nafamostat immediate-release tablet, the bioavailability is low compared to that of the reference drug, but the effective blood concentration that makes it an optimum choice for coronavirus treatment is several tens of ng/mL; hence, it was understood that there was no major problem. In addition, among oral formulations, immediate-release tablets showed significantly faster drug release and absorption than solution formulations, and the half-life of immediate-release tablets was increased by approximately 15 times or more compared to that of the literature report [45]. Furthermore, it was proven that bioavailability was increased by approximately 30% compared to that of solution formulations. Currently, in clinical trials of injections for severely ill patients, it is necessary to maintain a low blood concentration while slowly administering an infusion over 24 h. Accordingly, it was understood that it would be possible to maintain sufficient blood concentration when taking the tablet three times a day, and it was found that the immediate-release tablet is most suitable for the treatment of COVID-19.

## 4. Conclusions

Many reports have confirmed that the existing drug nafamostat mesylate effectively treats COVID-19. As a treatment for COVID-19, nafamostat mesylate injection, an existing anticoagulant/pancreatitis treatment, was developed as an immediate-release tablet. The optimized nafamostat immediate-release tablet composition was selected as nafamostat mesylate, microcelac, crospovidone, hydroxypropyl cellulose, and sodium stearyl fumarate, although the DoE and QbD approaches and the dissolution and stability were optimized only when manufactured through a direct tableting manufacturing process. To develop this COVID-19 tablet, risk-based PHA and FMEA were performed, and the binding agent and disintegrant, which are the most influential factors, were selected as the CMA, while the design space and optimal control range were established. In the stability study of the wet granulation process and the direct tableting process for approximately 2 months, the related substances in the wet granulation process showed a high value of approximately 0.1% to 1.8%, which was due to the interaction with the water used as the binding solution for wet granulation. It was determined that the related substances were rapidly generated after instability. Thus, it was determined that the direct tableting process, rather than the wet granulation process, was an appropriate process for nafamostat immediate-release tableting. Moreover, because a monkey PK study was conducted, it was confirmed that the bioavailability was as low as 5% or less compared to that of the reference drug, but the blood half-life was increased by approximately 15 times. Additionally, the bioavailability of the immediate-release tablet was approximately 25% higher than that of the liquid formulation, confirming its potential as a treatment for COVID-19. Therefore, the developed nafamostat immediate-release tablet is believed to be effective and widely commercialized as a treatment for the coronavirus. Lastly, the cost and time can be greatly reduced by utilizing drug recreation developed by identifying new indications for existing drugs.

## Figures and Tables

**Table 1:** Characteristics of nafamostat mesylate.

Nafamostat Mesylate			
Chemical structure	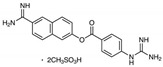	CAS No.	82956-11-4
		Chemical name	Nafamostat mesylate
		Formula	C_19_H_17_N_5_O_2_∙2CH_4_O_3_S
Mol. mass	539.6 g/mol	Description	White powder
Melting point	637.2 °C	Solubility	0.0341 mg/mL (in water)
Boiling point		Log *p*	1.91, 2.52
pKa	11.32	BCS class	
Storage condition	A light-shielding airtight container, stored at room temperature (1–30 °C)		
Mechanism of action	TMPRSS2 activity inhibitor. Nafamostat exhibits inhibitory action on trypsin; it also inhibits clotting factors such as thrombin, Xa, XIIa, VIIa, kallikrein, and complement, and inhibits platelet aggregation		
Pharmacokinetics	Half-life: 8 min		

**Table 2:** The solubility of nafamostat mesylate in various solvents (n = 3).

Solvent	Apparent	Equilibrium (mg/mL)	
		1 h	4 h
Water	++++	18.918 ± 0.047	18.825 ± 0.019
Ethanol	+	2.022 ± 0.046	2.201 ± 0.027
pH 1.2	+++	9.979 ± 0.003	8.558 ± 0.209
pH 2.0	+++	14.786 ± 0.040	16.463 ± 0.066
pH 3.0	+	0.002 ± 0.004	0.001 ± 0.002
pH 4.0	+++	15.470 ± 0.160	16.016 ± 0.226
pH 5.0	+	0.001 ± 0.034	0.000 ± 0.008
pH 6.0	+	0.025 ± 0.003	0.019 ± 0.004
pH 6.8	+	0.010 ± 0.003	0.009 ± 0.001
pH 7.0	+	0.008 ± 0.001	0.006 ± 0.002
pH 8.0	+	17.500 ± 0.014	15.371 ± 0.019
pH 9.0	+	0.096 ± 0.003	0.078 ± 0.005
pH 10.0	+	0.000 ± 0.001	0.000 ± 0.001
pH 11.0	+	0.004 ± 0.002	0.008 ± 0.004
pH 12.0	+	0.000 ± 0.002	0.042 ± 0.009

+: practically insoluble, ++: very slightly soluble, +++: slightly soluble, ++++: sparingly soluble, +++++: soluble, ++++++: easily soluble, +++++++: very soluble.

**Table 3:** Total impurities of compatibility test between nafamostat mesylate and each excipient via HPLC (%).

Items	Total Impurities		
	Initial (%)	40 °C/60% RH 4 Weeks (%)	60 °C/75% RH 4 Weeks (%)
Nafamostat mesylate	0.04 ± 0.01	0.10 ± 0.03	0.13 ± 0.01
Mannitol	0.04 ± 0.01	0.09 ± 0.03	0.14 ± 0.02
Lactose	0.07 ± 0.03	0.10 ± 0.01	0.13 ± 0.01
Dicalcium phosphate dihydrate	0.04 ± 0.02	3.20 ± 0.01	1.26 ± 0.03
Microcrystalline cellulose	0.05 ± 0.01	0.09 ± 0.01	0.12 ± 0.01
Pregelatinized starch	0.05 ± 0.01	0.09 ± 0.01	0.13 ± 0.02
Precipitated calcium carbonate	0.07 ± 0.01	0.16 ± 0.01	0.28 ± 0.02
Sodium starch glyconate	0.06 ± 0.01	0.15 ± 0.02	0.23 ± 0.01
Crospovidone	0.05 ± 0.01	0.10 ± 0.02	0.12 ± 0.03
Sodium croscamellose	0.03 ± 0.01	0.11 ± 0.04	0.12 ± 0.01
Sodium stearyl fumarate	0.03 ± 0.05	0.12 ± 0.03	0.15 ± 0.02
Magnesium stearate	0.03 ± 0.02	0.13 ± 0.01	0.20 ± 0.01
Povidone K	0.05 ± 0.01	0.10 ± 0.01	0.14 ± 0.03
Hydroxypropyl methylcellulose	0.05 ± 0.01	0.05 ± 0.03	0.16 ± 0.05
Hydroxypropyl cellulose	0.05 ± 0.03	0.08 ± 0.08	0.14 ± 0.04
Calcium hydroxide	1.08 ± 0.01	0.64 ± 0.01	9.00 ± 0.01
Sodium hydroxide	0.32 ± 0.04	3.82 ± 0.01	9.05 ± 0.01

**Table 4:** (a) The stability of wet granulation and direct compression and (b) stability according to the ratio of excipients for 1 month.

**(a)**							
**Process Method**	**Initial (%)**	**1 M**			**2 M**		
		**RT (%)**	**AC (%)**	**HC (%)**	**RT (%)**	**AC (%)**	**HC (%)**
Wet granulation	0.05 ± 0.02	0.25 ± 0.02	1.53 ± 0.01	6.16 ± 0.05	0.25 ± 0.02	2.10 ± 0.01	9.35 ± 0.01
Direct compression	0.06 ± 0.01	0.05 ± 0.01	0.11 ± 0.01	0.95 ± 0.04	0.11 ± 0.02	0.33 ± 0.01	3.04 ± 0.01
**(b)**							
**Direct Compression**	**Initial (%)**	**1 M**			**2 M**		
		**RT (%)**	**AC (%)**	**HC (%)**	**RT (%)**	**AC (%)**	**HC (%)**
100/300 mg	0.06 ± 0.02	0.06 ± 0.02	0.07 ± 0.02	0.19 ± 0.02	0.11 ± 0.02	0.23 ± 0.02	0.34 ± 0.02
100/500 mg	0.05 ± 0.02	0.05 ± 0.02	0.11 ± 0.02	0.95 ± 0.02	0.11 ± 0.02	0.33 ± 0.02	3.04 ± 0.02

Abbreviations: RT, room temperature; AC, accelerated condition; HC, hard condition; w, week.

**Table 5:** (a) Qualitative preliminary hazard analysis (PHA) and (b) quantitative failure mode effects analysis (FMEA) of material attributes (MAs).

**(** **a)**									
**CQAs** **^1^**	**Nafamostat Mesylate**	**Diluent**	**Binder**	**Disintegrant**	**Anti-Adherent**	**Glidant**		**Lubricant**	
**Physical**	Low	Low	Low	Low	Low	Low		Low	
**Assay**	Low	Low	Low	Low	Low	Low		Low	
**Uniformity**	Low	Low	Medium	Low	Low	Low		Medium	
**Impurities**	Low	Low	Low	Low	Low	Low		Low	
**Dissolution**	Low	Low	High	High	Low	Low		Medium	
**(b)**									
**Functions**	**CMAs** **^2^**	**Failure Mode** **(Critical Event)**	**Effect on CQAs with Respect to QTPP ^3^** **(Justification of Failure Mode)**			**P** **^4^**	**S** **^5^**	**D** **^6^**	**RPN** **^7^**
Physical property of API ^8^	Solid state form	Different crystal form/Different form	The solubility of active pharmaceutical ingredient (API) may be affected, and the dissolution of the drug product is affected, thus causing damage to bioavailability and efficacy			1	2	2	4
Chemical property of API	Solubility	Different Salt/ Different form	May affect the dissolution of tablets; thus, bioavailability and efficacy may be compromised			1	1	2	2
	Impurity in the manufacturing process	Low purity	May affect the assay and impurity of tablets; thus, quality and safety may be compromised			1	2	2	4
	Chemical stability	Unstable	Decomposition products may be affected by dry hear/oxidation/hydrolysis/UV light, thus causing quality and safety damage			1	1	2	2
Diluent	PSD ^9^	Uneven	It can affect the flow properties of blending and can affect the content uniformity; thus, quality/safety may be compromised			1	1	2	2
	Moisture content	High	May affect the impurity profile, thus causing damage to safety			3	2	2	12
Binder	Volume of binder	Higher than optimum	Produces hard mixtures, which can affect disintegration and dissolution time; thus, bioavailability and efficacy may be compromised			4	3	3	**36**
		Lower than optimum	Loose, fragile mixtures can produce tablets of weaker hardness (fast disintegration); thus, bioavailability and efficacy may be compromised			4	3	3	**36**
Disintegrant	Concentration of disintegrant	Higher than optimum	The desired dissolution pattern cannot be obtained, and the hardness of the tablet may be affected; thus, bioavailability and efficacy may be compromised			4	4	3	**48**
		Lower than optimum	The desired dissolution pattern cannot be obtained; thus, bioavailability and efficacy may be compromised			4	3	3	**36**
Anti-adherent	Concentration of anti-adherent	Lower than optimum	It may be difficult to discharge tablets from tooling; the excipient can be stuck on the surface of the filing die; thus, product quality may be compromised			3	3	2	18
Glidant	Concentration of glidant	Lower than optimum	By reducing the friction in the particles, it may affect the flowability of granules or powders such as die friction; may affect content uniformity; thus, content uniformity and product quality may be compromised			2	2	2	8
Lubricant	Concentration of lubricant	Higher than optimum	Hydrophobic lubricants can be coated on the surface of drug particles, which can delay dissolution; thus, efficacy may be compromised			3	3	3	27
		Lower than optimum	The powder can stick to the surface of tooling/punch and cause picking; thus, product quality may be compromised			3	3	3	27

Green: a wide range of acceptable risks; yellow: acceptable risk; red: unacceptable risk. ^1^ CQAs: critical quality attributes. ^2^ CMAs: critical material attributes. ^3^ QTPP: quality target product profiles. ^4^ P: probability. ^5^ S: Severity. ^6^ D: detectability. ^7^ RPN: risk priority number, if the total RPN score is more than 30 (marked red), to be prepared for risks, major actions such as DOE must be performed. ^8^ API: active pharmaceutical ingredient. ^9^ PSD: particle size distribution.

**Table 6:** Critical material attributes (CMAs) affecting critical quality attributes (CQAs).

Run	CMAs		CQAs		
	X1 HPC (%)	X2 Crospovidone (%)	Y1 Hardness (%)	Y2 Disintegration (%)	Y3 Friability (%)
1	1.0	1.0	1.8	19.3	1.3
2	3.0	1.0	18.4	28.3	0.2
3	1.0	5.0	5.9	4.0	1.3
4	3.0	5.0	18.6	18.0	0.3
5	0.6	3.0	0.9	6.	1.5
6	3.4	3.0	16.3	18.0	0.1
7	2.0	0.2	8.8	28.3	0.3
8	2.0	3.0	16.8	4.0	0.3
9	2.0	3.0	17.1	8.5	0.2
10	2.0	3.0	16.5	7.9	0.1
11	2.0	3.0	16.9	8.0	0.2
12	2.0	3.0	17.6	9.1	0.3
13	2.0	3.0	17.0	8.5	0.3

**Table 7:** PK study of reference drug and test drug: pharmacokinetics parameters.

Parameter	Reference Drug	Test Drug	
	Injection (10 mg/Head, IV)	Solution (200 mg/Head, PO)	IR Tablet (200 mg Tb/Head, PO)
AUC_last_ (ng·h/mL)	2182.1 ± 32.7	898.6 ± 76.2	1140.5 ± 291.7
C_max_ (ng/mL)	11,108.0 ± 1340.7	216.1 ± 69.2	327.3 ± 144.3
T_max_ (h)	0.02 ± 0.0	4.0 ± 0.0	1.00 ± 1.3
t_1/2_ (h)	1.5 ± 0.0	1.9 ± 0.9	2.2 ± 1.1
Cl_inf_/F	4.2 ± 0.02	197.2 ± 34.7	160.1 ± 45.9

Reference drug, nafamostat mesylate injection; test drug, nafamostat mesylate liquid; IR tablet.

## Data Availability

The data presented in this study are included in this published article.
